# Supplementary material for: Mechanism of Ψ-Pro/C-degron recognition by the CRL2FEM1B ubiquitin ligase
Source: Nat Commun. 2024 Apr 26;15:3558. doi: 10.1038/s41467-024-47890-5 (PMC11053023; doi:10.1038/s41467-024-47890-5)
Supplement: Supplementary file 1 — Supplementary Information [file 41467_2024_47890_MOESM1_ESM.pdf]

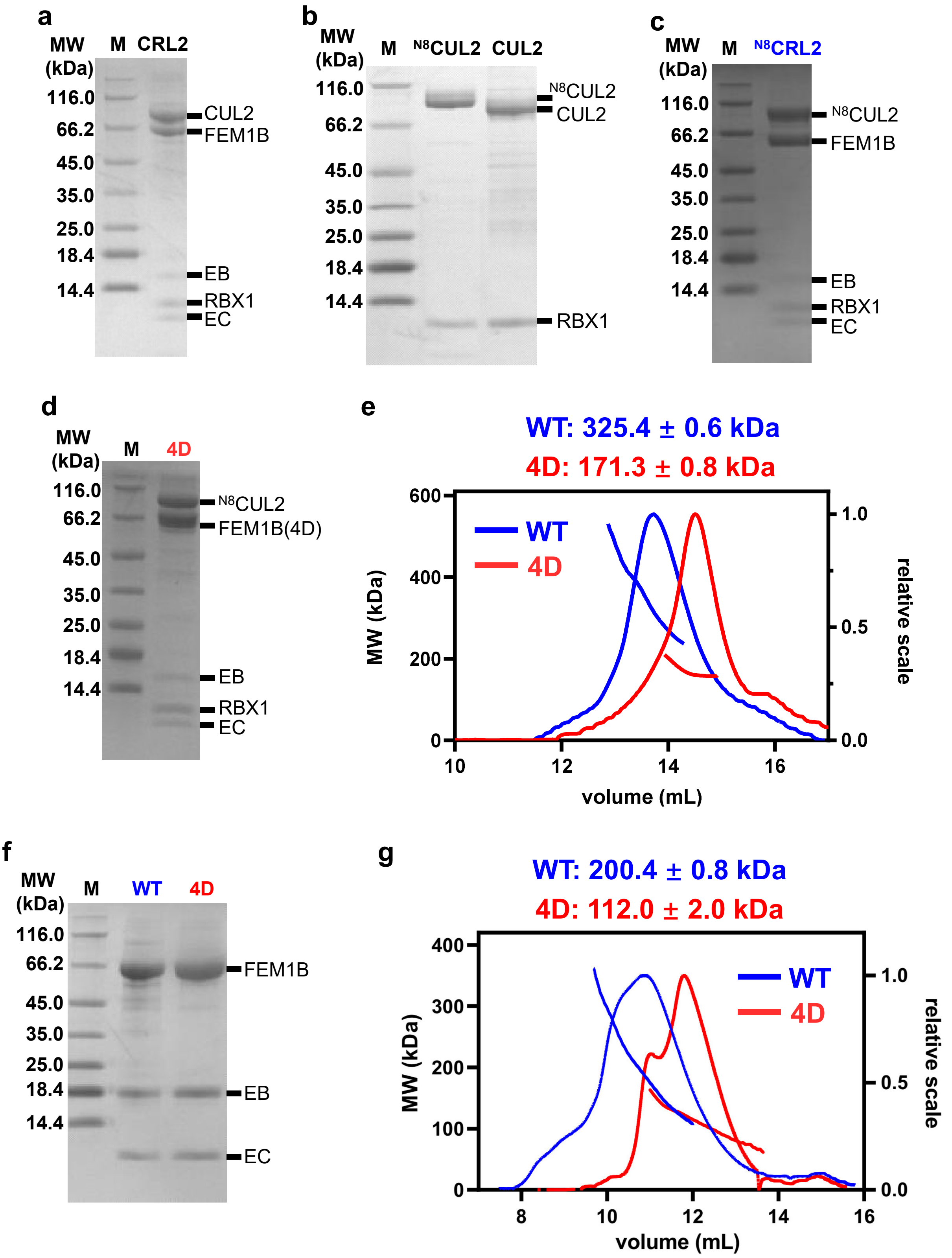

Supplementary Fig. 1. Purification of recombinant CRL2^{FEM1B} complexes. **a-d** The SDS-PAGE gels of purified unmodified CRL2^{FEM1B} complex (a), unmodified and neddylated CUL2-RBX1 complex (b), neddylated CRL2^{FEM1B} complex (c), and neddylated mutant CRL2^{FEM1B} complex containing the FEM1B mutant F549D/V584D/I587D/L588D (4D) (d). **e** Static light scattering (SLS) experiments indicate that the peaks of ^{N8}CRL2^{FEM1B} (WT) and ^{N8}CRL2^{FEM1B} (4D mut) correspond to the molecular weights of 325 and 171 kDa, respectively. **f** The SDS-PAGE gel of FEM1B-EB-EC complexes (WT and 4D mutant). **g** Static light scattering (SLS) experiments indicate that the peaks of WT and 4D mutant FEM1B-EB-EC correspond to the molecular weights of 200 and 112 kDa, respectively.

Supplementary Figure 1

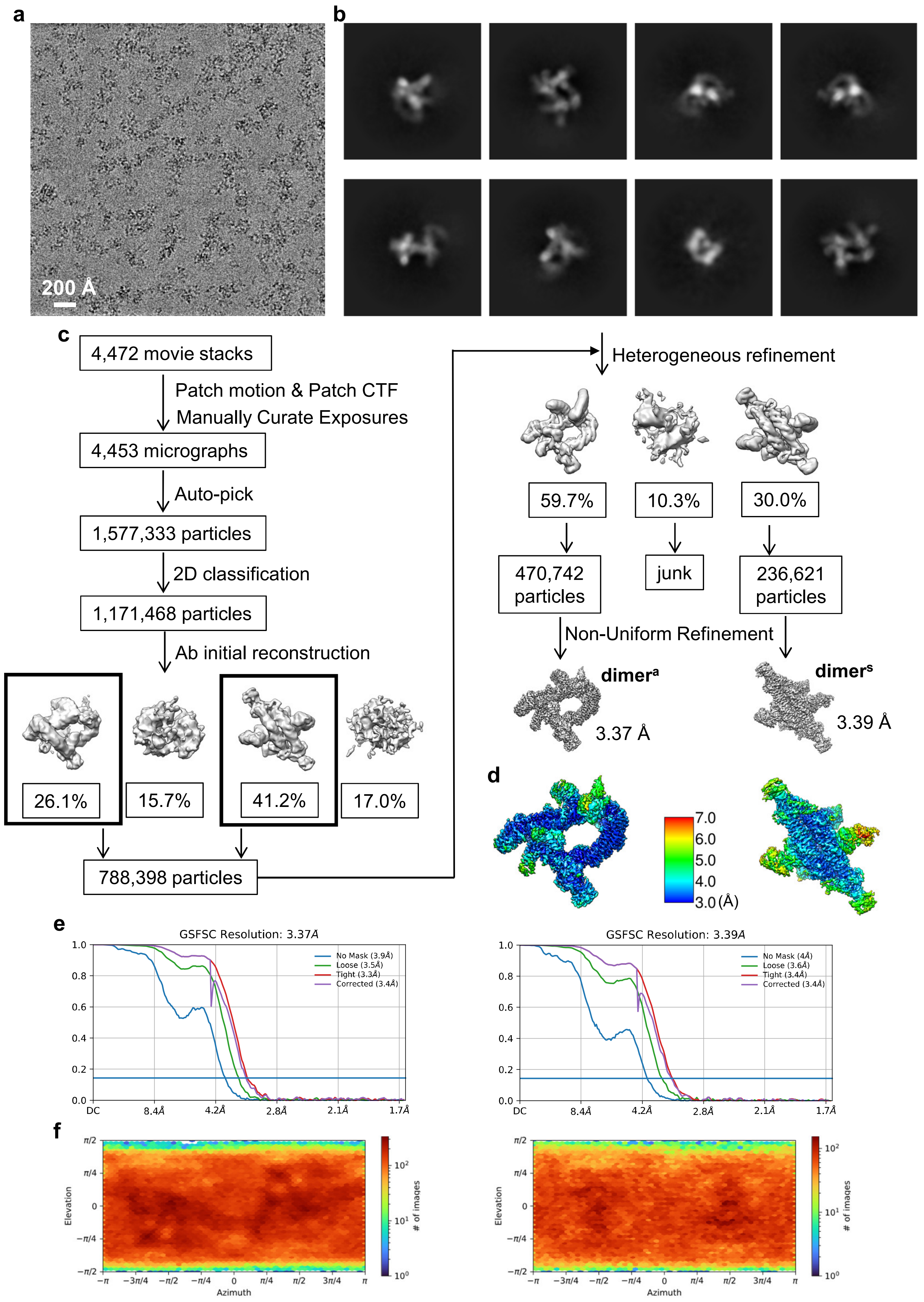

Supplementary Figure 2

Supplementary Fig. 2. Single-particle cryo-EM analysis of the unmodified CRL2^{FEM1B} complex with the CCDC89 C-degron. **a** Representative motion-corrected cryo-EM micrograph. **b** Reference-free 2D class averages. **c** Workflow of the data processing. **d** Resolution maps for the final 3D reconstructions of dimer^{a} (left) and dimer^{s} (right). **e** Gold standard FSC plots for the 3D reconstructions of dimer^{a} (left) and dimer^{s} (right), calculated in cryoSPARC. **f** Euler angle distribution of the particle images.

Supplementary Figure 2

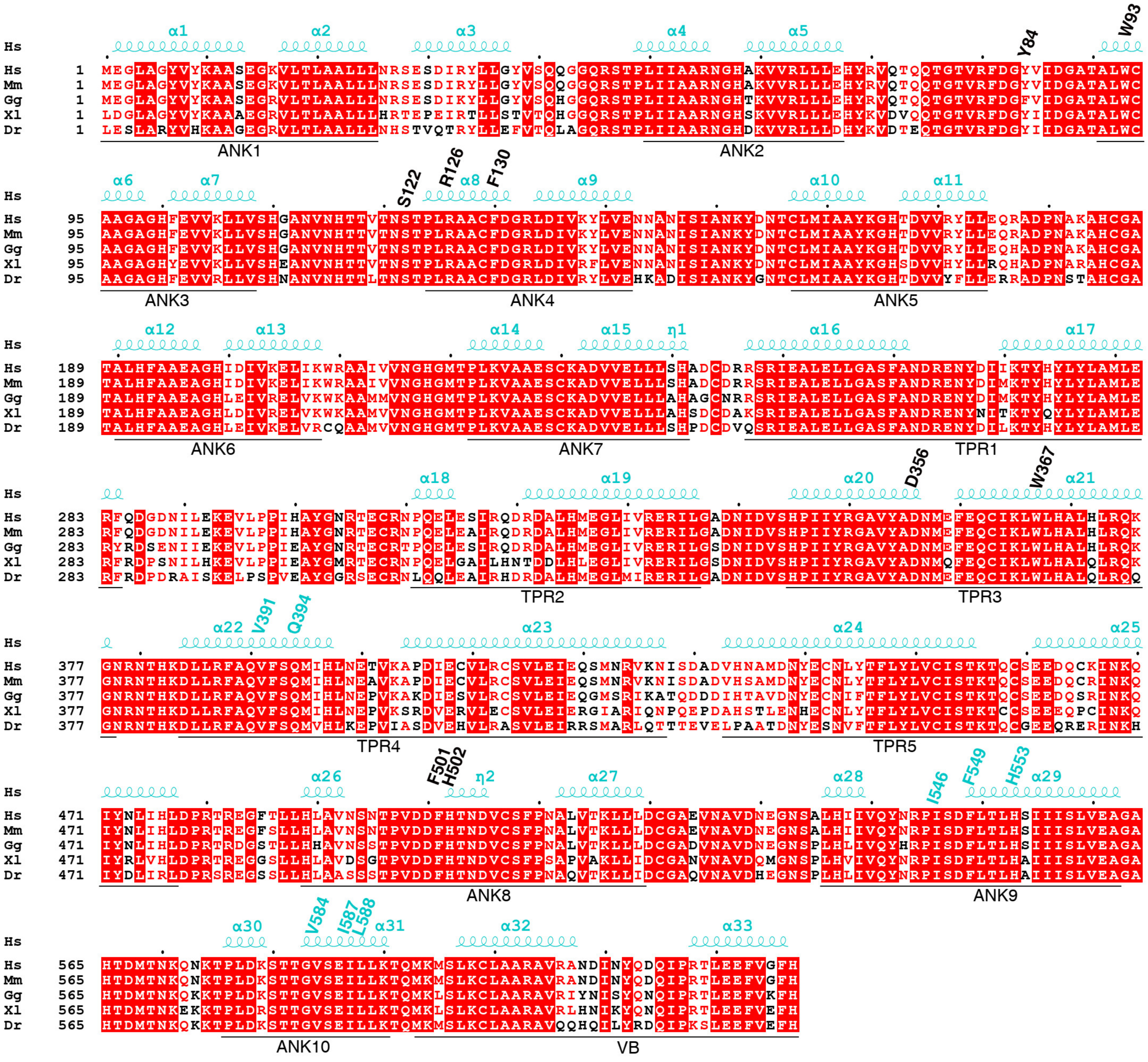

Supplementary Figure 3. Sequence alignment of human FEM1B and its orthologs from different species, including those from Homo sapiens (Hs, NP_056137.1), Mus musculus (Mm, NP_034323.1), Gallus gallus (Gg, NP_001025724.1), Xenopus laevis (Xl, NP_001085685.1), and Danio rerio (Dr, XP_695502.4). The secondary structures of human FEM1B are labelled at the top of the sequences. The ankyrin repeats (ANK), TPR repeats, and the VHL box (VB) of FEM1B are shown at the bottom. FEM1B residues involved in recognizing P-1 and H-21 of CCDC89 are indicated in black, and the FEM1B residues involved in dimerization are indicated in cyan.

Supplementary Figure 3

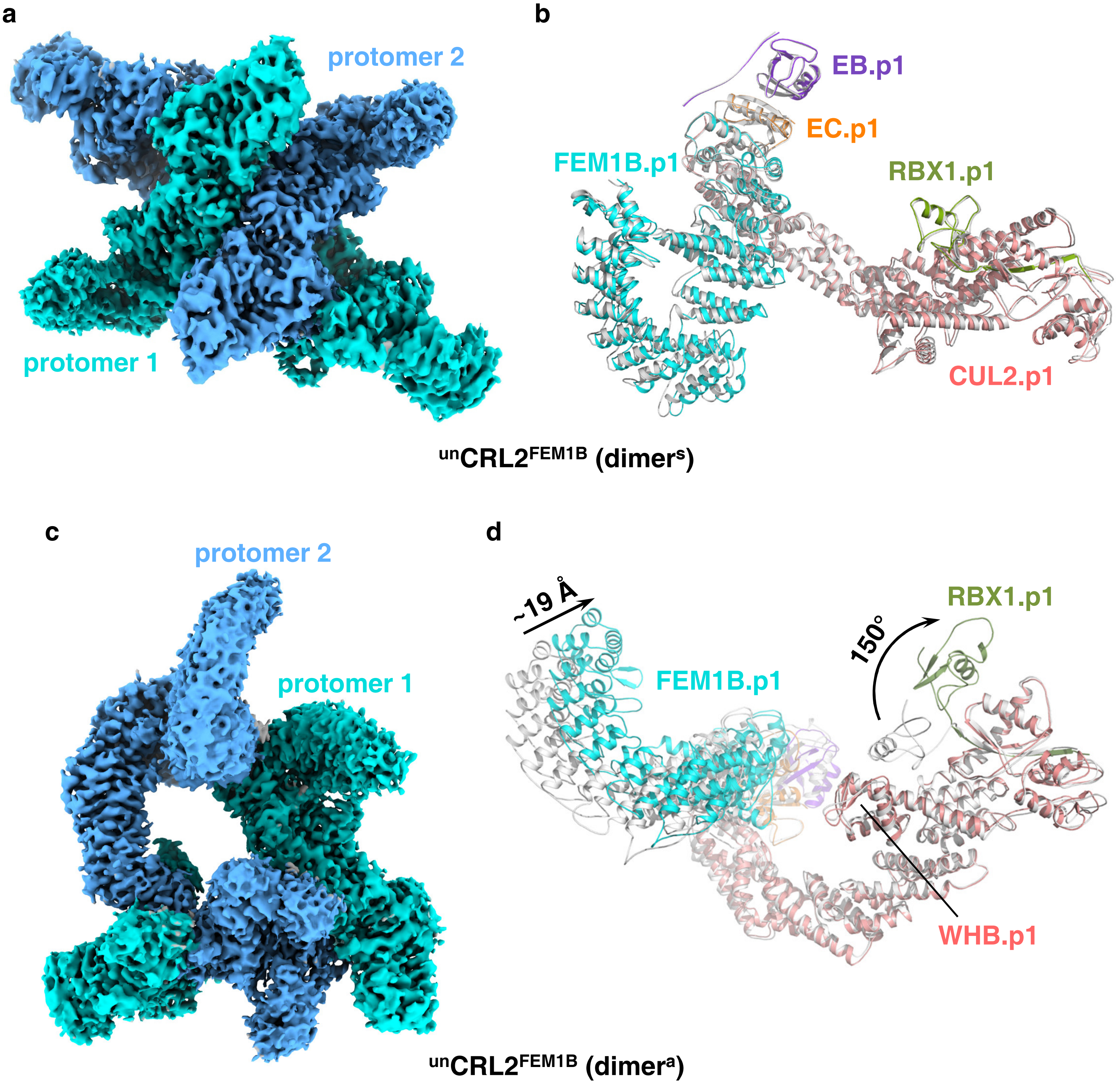

Supplementary Figure 4. Architecture of ^{un}CRL2^{FEM1B}. **a** architecture of ^{un}CRL2^{FEM1B} dimer^{s} with the two protomers colored in cyan and blue. **b** The two protomers of ^{un}CRL2^{FEM1B} dimer^{s} were were superimposed, with the protomer 1 colored by subunits and the protomer 2 in grey. **c** architecture of ^{un}CRL2^{FEM1B} dimer^{a} with the two protomers colored in cyan and blue. **d** The two protomers of ^{un}CRL2^{FEM1B} dimer^{a} were were superimposed, with the protomer 1 colored by subunits and the protomer 2 in grey.

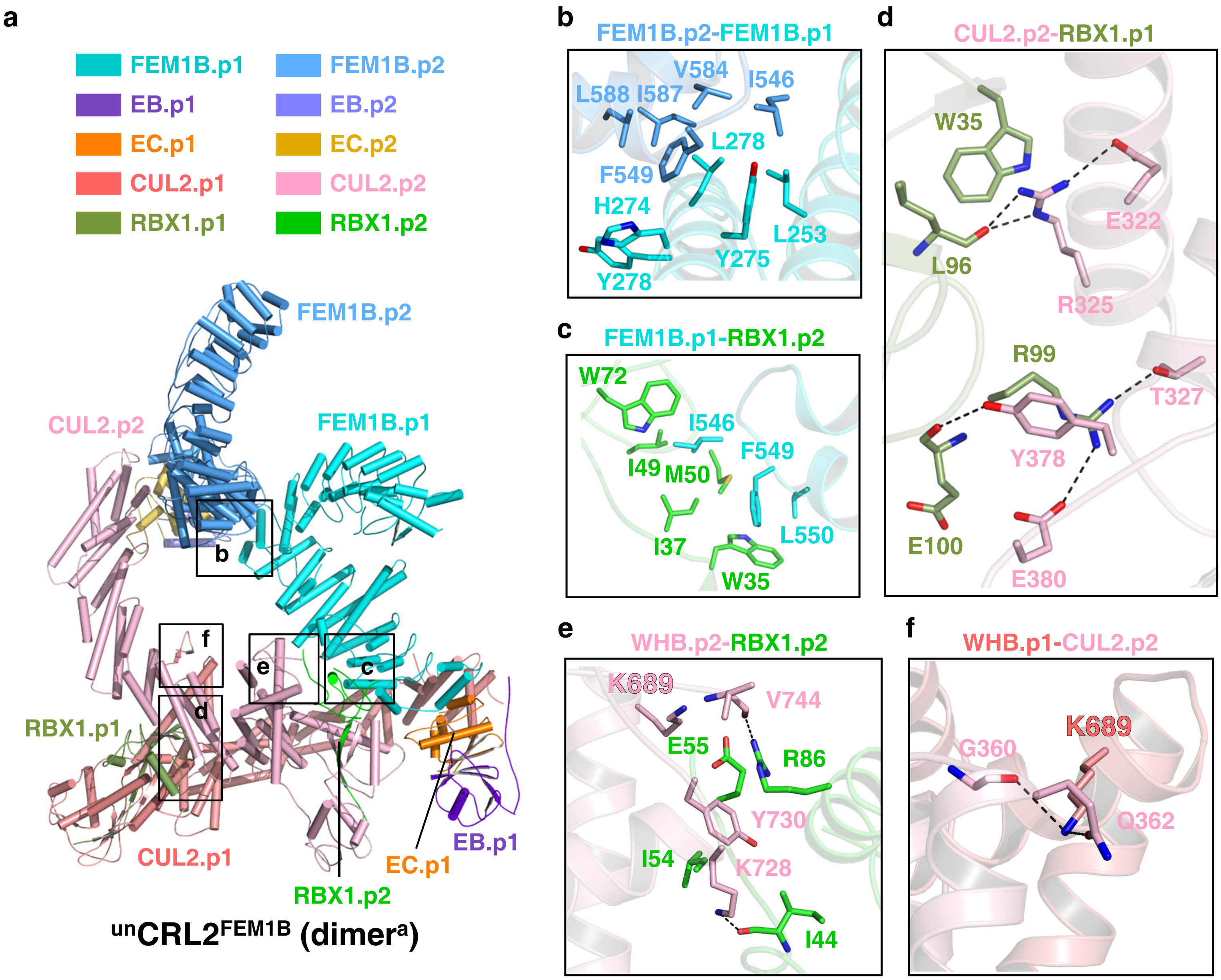

Supplementary Figure 5. Structure of unmodified CRL2^{FEM1B} dimer^{a}. **a** Overall structure of the ^{un}CRL2^{FEM1B} dimer^{a} shown in cartoon representation and colored by different subunits. **b-f** Close-up views of the interfaces of FEM1B.p2-FEM1B.p1 (b), FEM1B.p1-RBX1.p2 (c), CUL2.p2-RBX1.p1 (d), WHB.p2-RBX1.p1 (e), and WHB.p1-CUL2.p2 (f).

Supplementary Figure 5

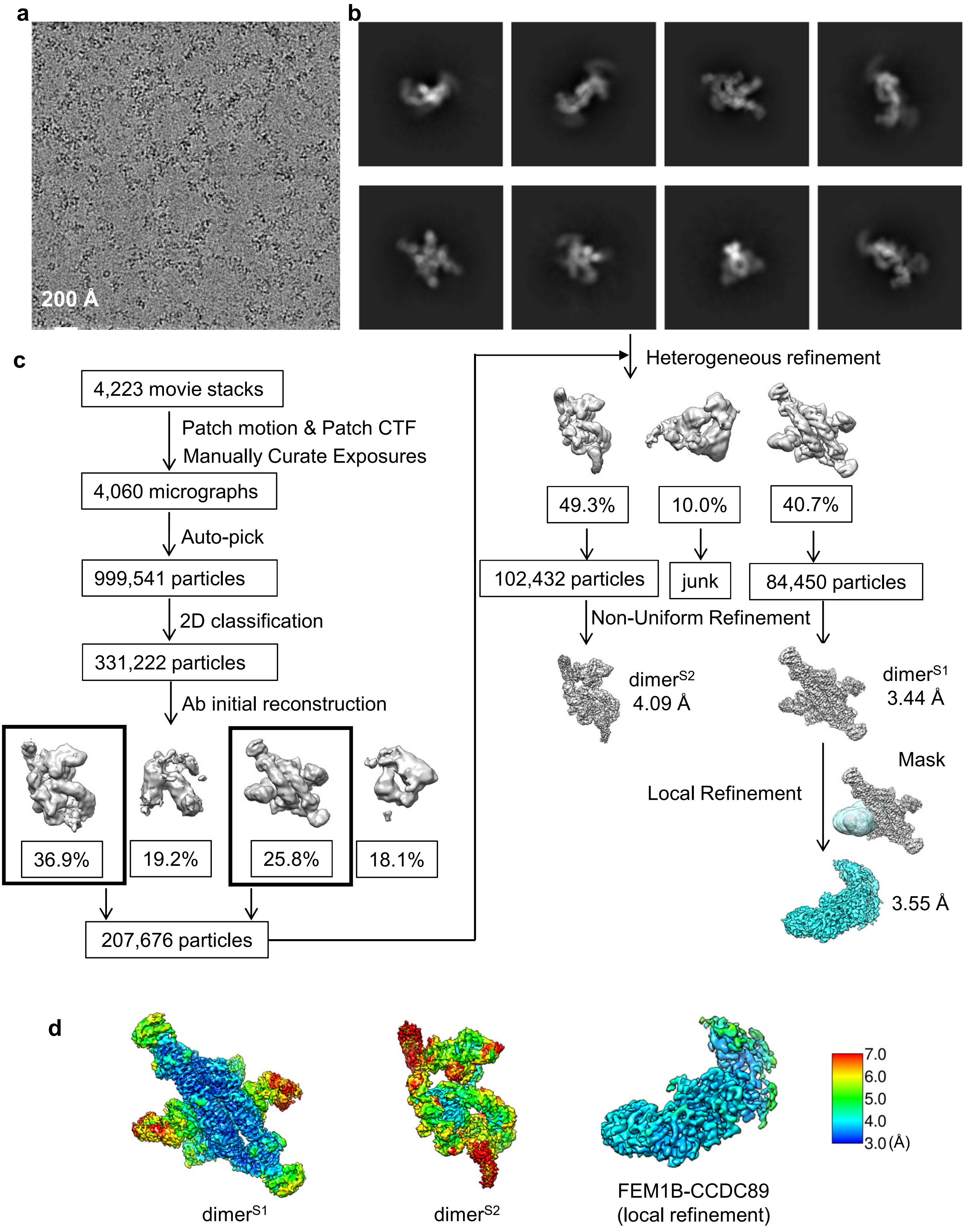

Supplementary Figure 6

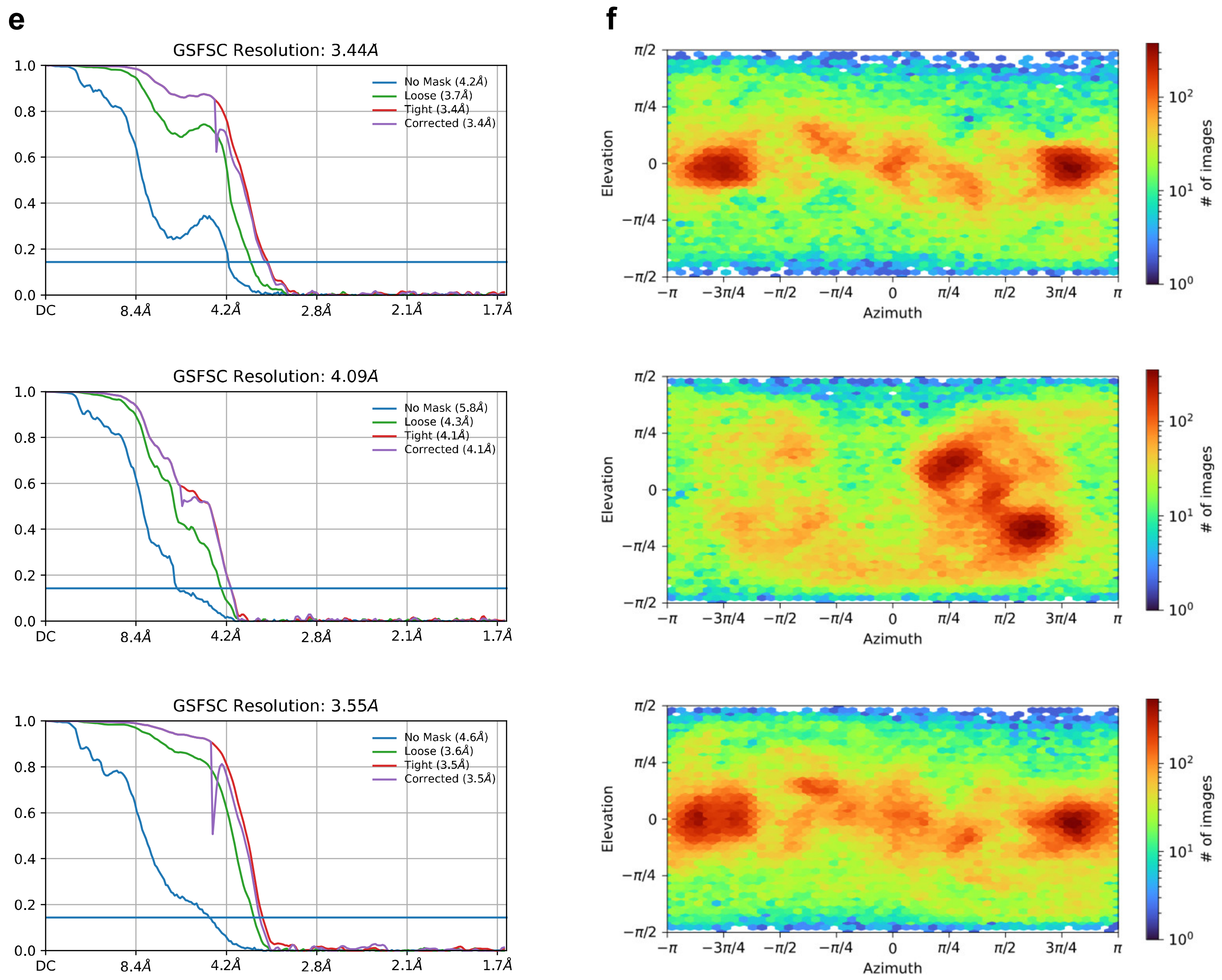

Supplementary Figure 6. Single-particle cryo-EM analysis of the neddylated CRL2^{FEM1B} complex with the CCDC89 C-degron. **a** Representative motion-corrected cryo-EM micrograph. **b** Reference-free 2D class averages. **c** Workflow of the data processing. **d** Resolution maps for the final 3D reconstructions of dimer^{S1} (left), dimer^{S2} (middle), and the FEM1B-CCDC89 complex (right). **e** Gold standard FSC plots for the 3D reconstructions of dimer^{S1} (upper), dimer^{S2} (middle), and the FEM1B-CCDC89 complex (bottom), calculated in cryoSPARC. **f** Euler angle distribution of the particle images.

Supplementary Figure 6

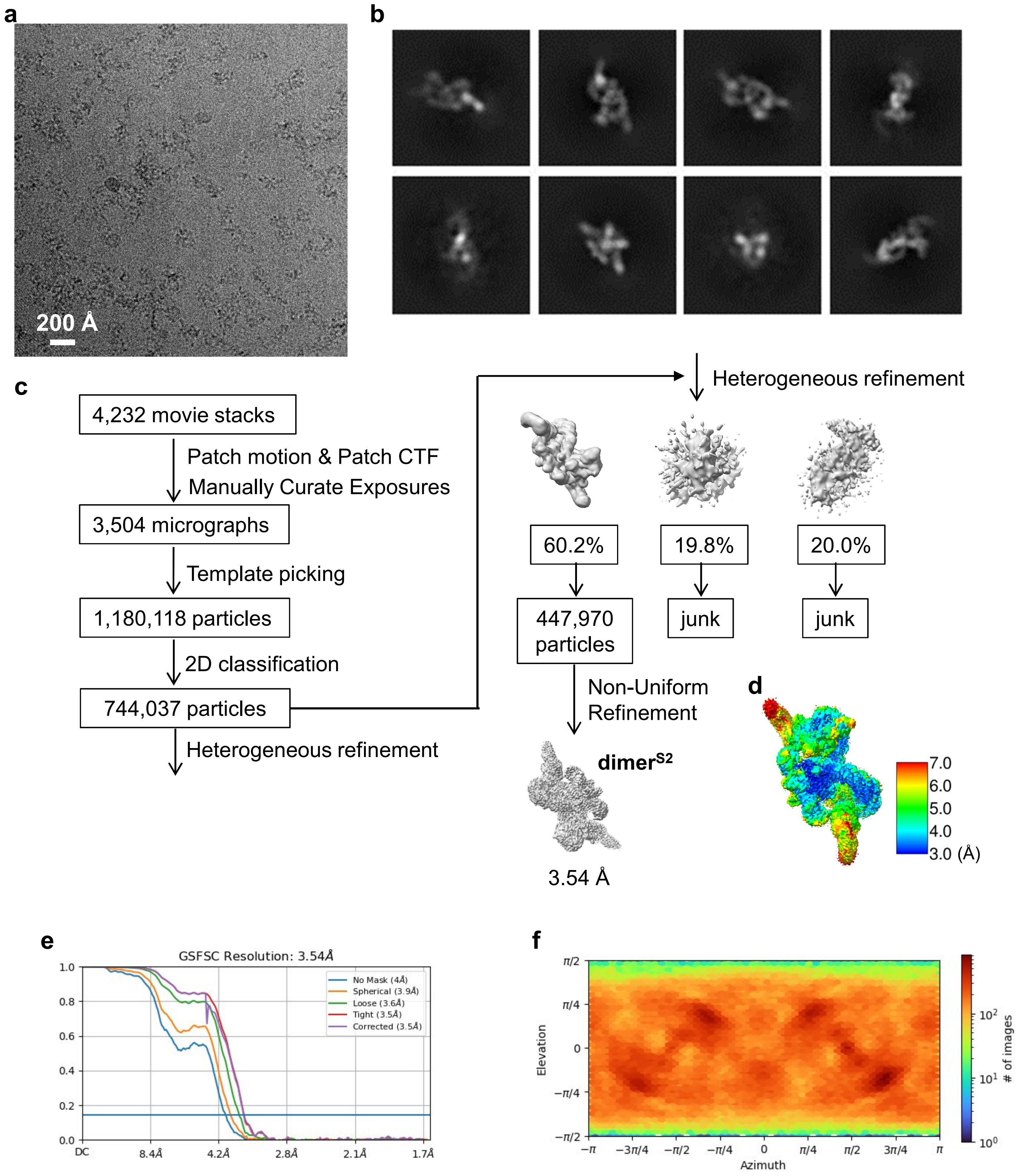

Supplementary Figure 7. Single-particle cryo-EM analysis of the neddylated CRL2^{FEM1B} complex with the CDK5R1 C-degron. **a** Representative motion-corrected cryo-EM micrograph. **b** Reference-free 2D class averages. **c** Workflow of the data processing. **d** Resolution maps for the final 3D reconstructions of dimer^{S2} (left). **e** Gold standard FSC plots for the 3D reconstructions of dimer^{S2}, calculated in cryoSPARC. **f** Euler angle distribution of the particle images.

Supplementary Figure 7

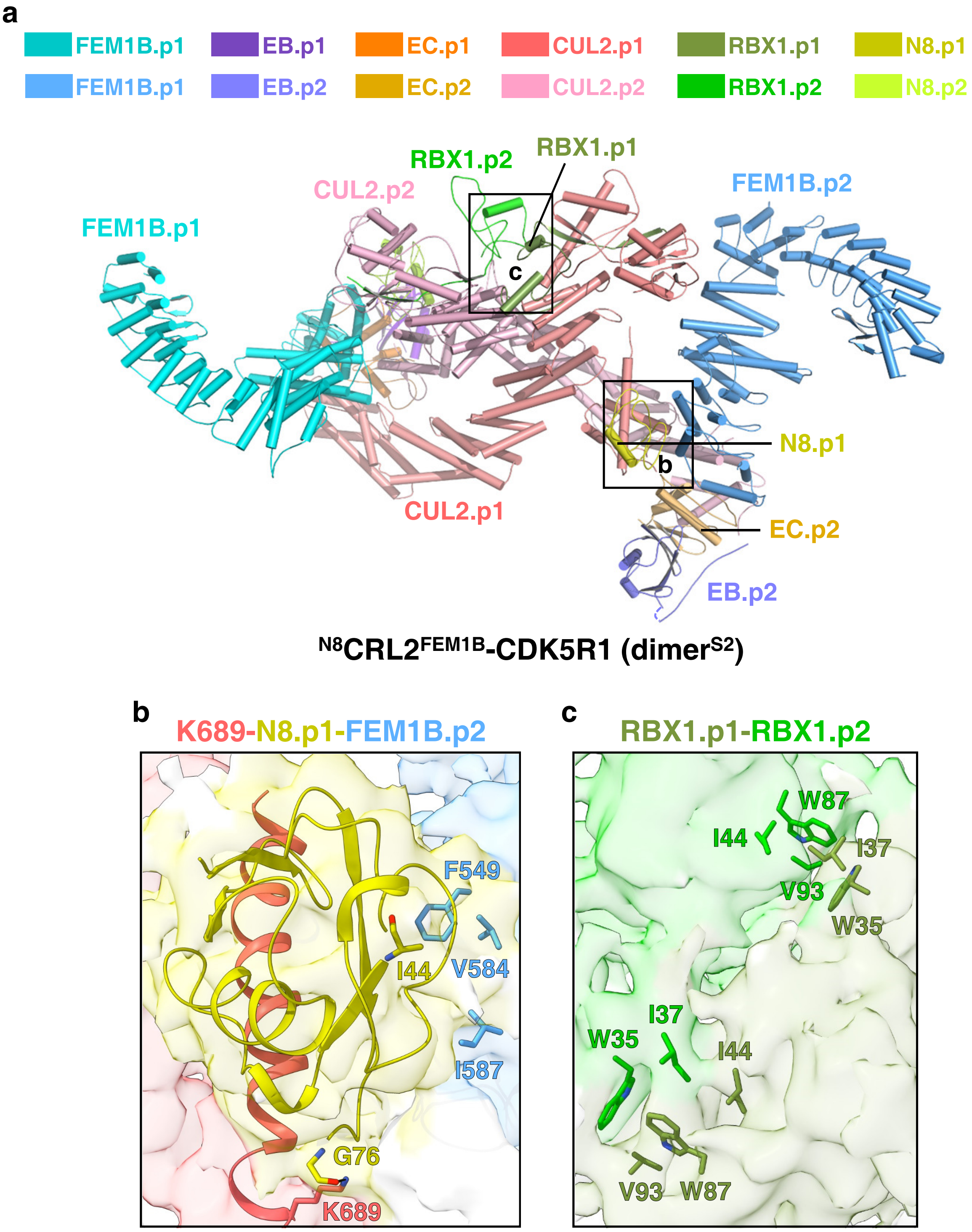

Supplementary Figure 8. Structure of neddylated CRL2^{FEM1B} dimer^{S2}. **a** Overall structure of the ^{N8}CRL2^{FEM1B} dimer^{S2} bound with the CDK5R1 C-degron. The structure is shown in cartoon representation and colored by different subunits. **b-c** Close-up views of the N8.p1-FEM1B.p2 interface with Lys689 of CUL2 neddylated (b), and the RBX1.p1-RBX1.p2 interface (c).

Supplementary Figure 8

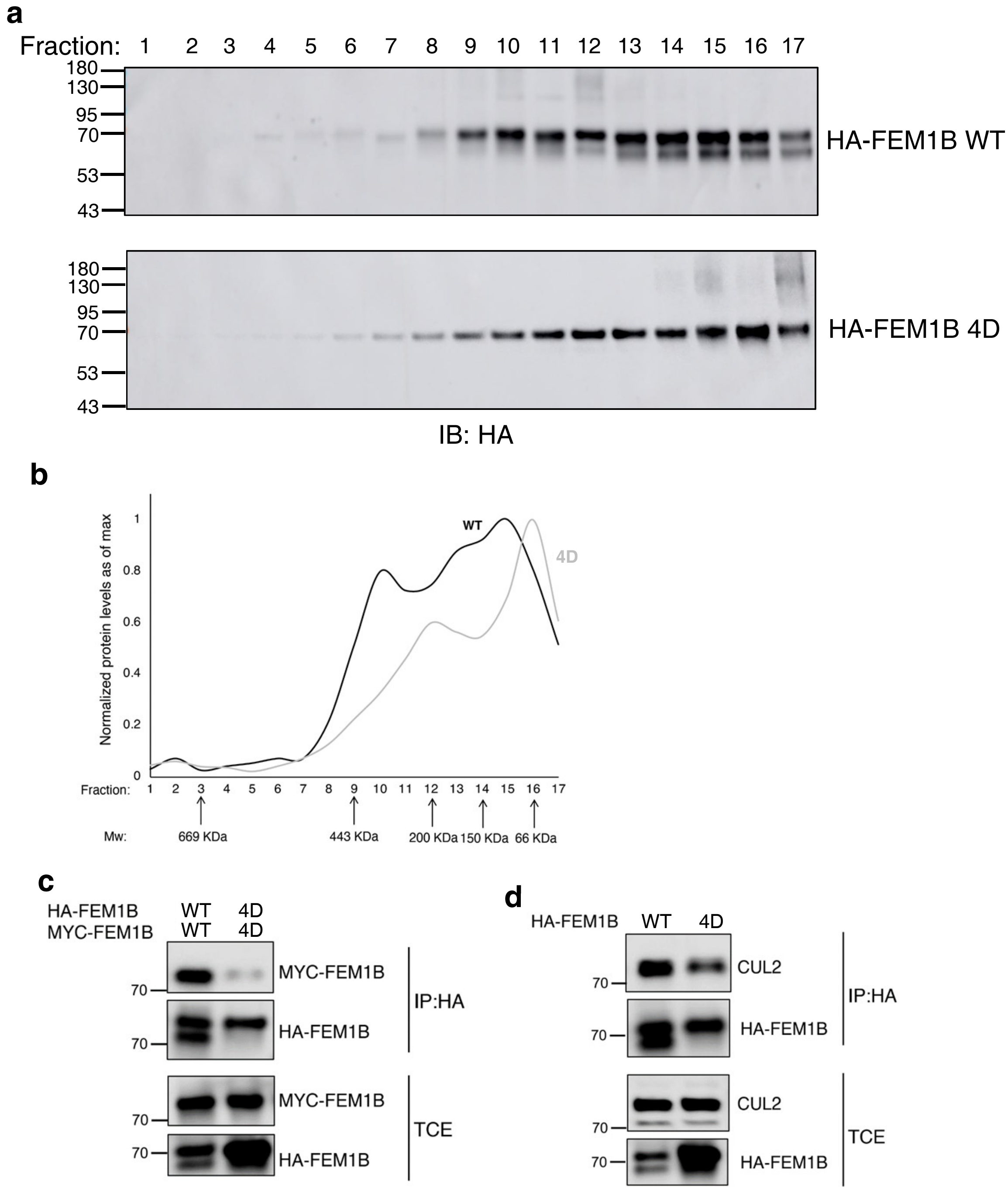

Supplementary Figure 9. CRL2^{FEM1B} dimerizes *in vivo.* (a-b) Analysis of FEM1B oligomerization by gel filtration chromatography. Lysates were isolated from FEM1B KO cells stably expressing HA-tagged FEM1B WT or the 4D mutant. **a** Eluted fractions from size-exclusion chromatography were separated by SDS-PAGE and analyzed by immunoblotting (IB) with an anti-HA antibody. Gel filtration analysis of lysates extracted from FEM1B KO cells stably expressing HA-FEM1B, WT or 4D mutant. **b** Quantification of FEM1B complexes by gel filtration. The gel filtration peaks corresponding to FEM1B WT and 4D mutant complexes from panel (a) are shown here after normalization to the peak containing the highest level of FEM1B. **c** HEK293T FEM1B KO cells co-expressing HA/MYC-FEM1B (WT or 4D) were immunoprecipitated (IP) with anti-HA beads. Immunoblot with anti-HA and anti-MYC antibodies detects FEM1B proteins in IP and total cell extract (TCE). WT HA-FEM1B co-IP more WT MYC-FEM1B compared to the IP performed in 4D mutant expressing cells. **d** HA-FEM1B (WT or 4D mutant) was immunoprecipitated (IP) from HEK293T FEM1B KO cells that stably express the HA-FEM1B variants. The isolated proteins were then analyzed by SDS-PAGE and immunoblotted with anti-HA and CUL2 antibodies. Source data are provided as a Source Data file.

Supplementary Figure 9

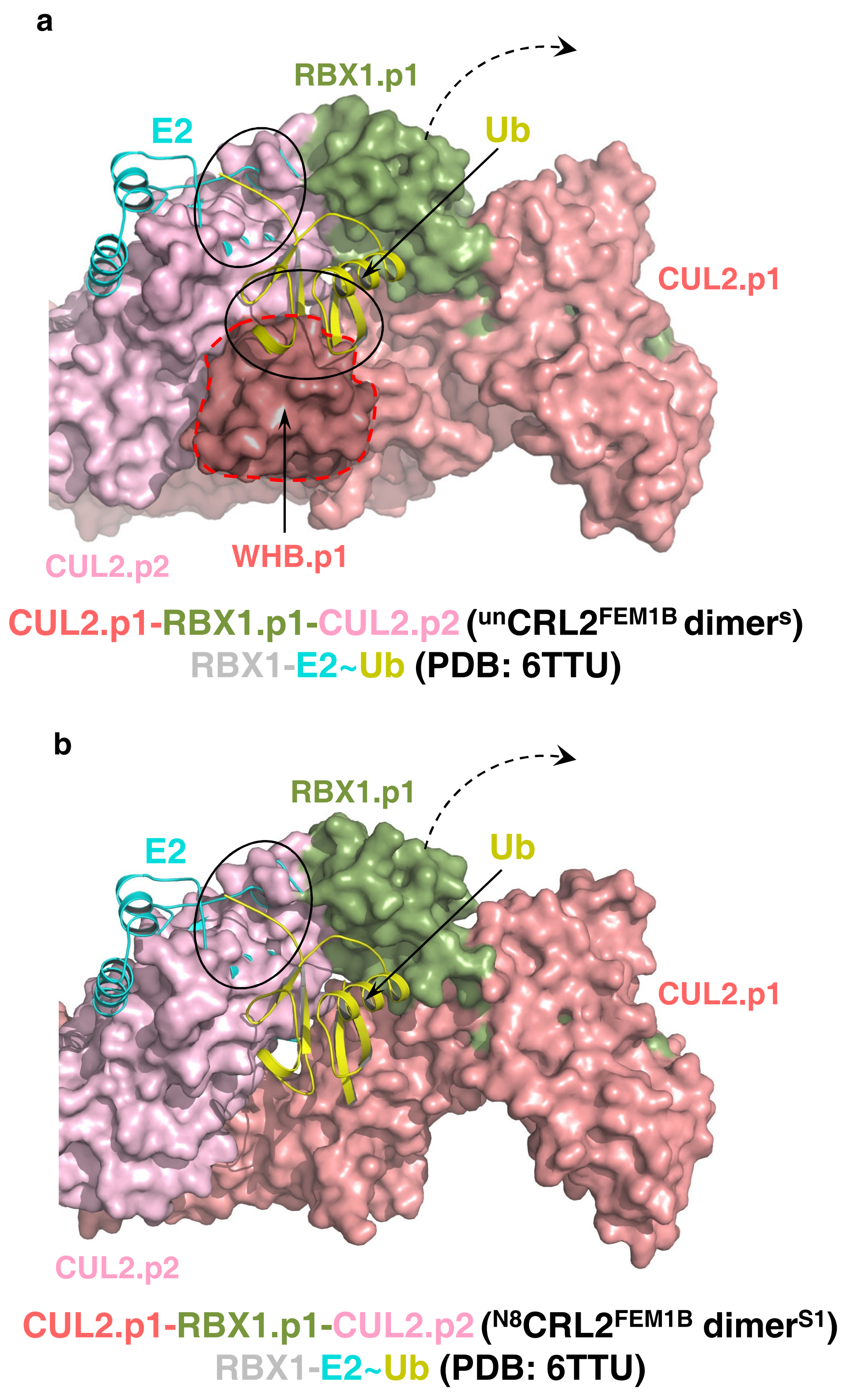

Supplementary Figure 10. NEDD8-mediated activation of CRL2^{FEM1B}. **a** The structure of ^{un}CRL2^{FEM1B} dimers was superimposed with that of the RBX1-E2~Ub complex (PDB: 6TTU), with the ^{un}CRL2^{FEM1B} dimers shown in surface representation and the RBX1-E2~Ub complex in cartoon. The CUL2.p1 WHB domain is highlighted to indicate the potential steric clash with Ub. The movement of RBX1.p1 upon binding to E2~Ub is indicated by the black dashed arrow. **b** The structure of ^{N8}CRL2^{FEM1B} dimer^{S1} was superimposed with that of the RBX1-E2~Ub complex. RBX1.p1 accessibility to E2~Ub would be enhanced in the absence of WHB domain of CUL2.p1.

Supplementary Figure 10

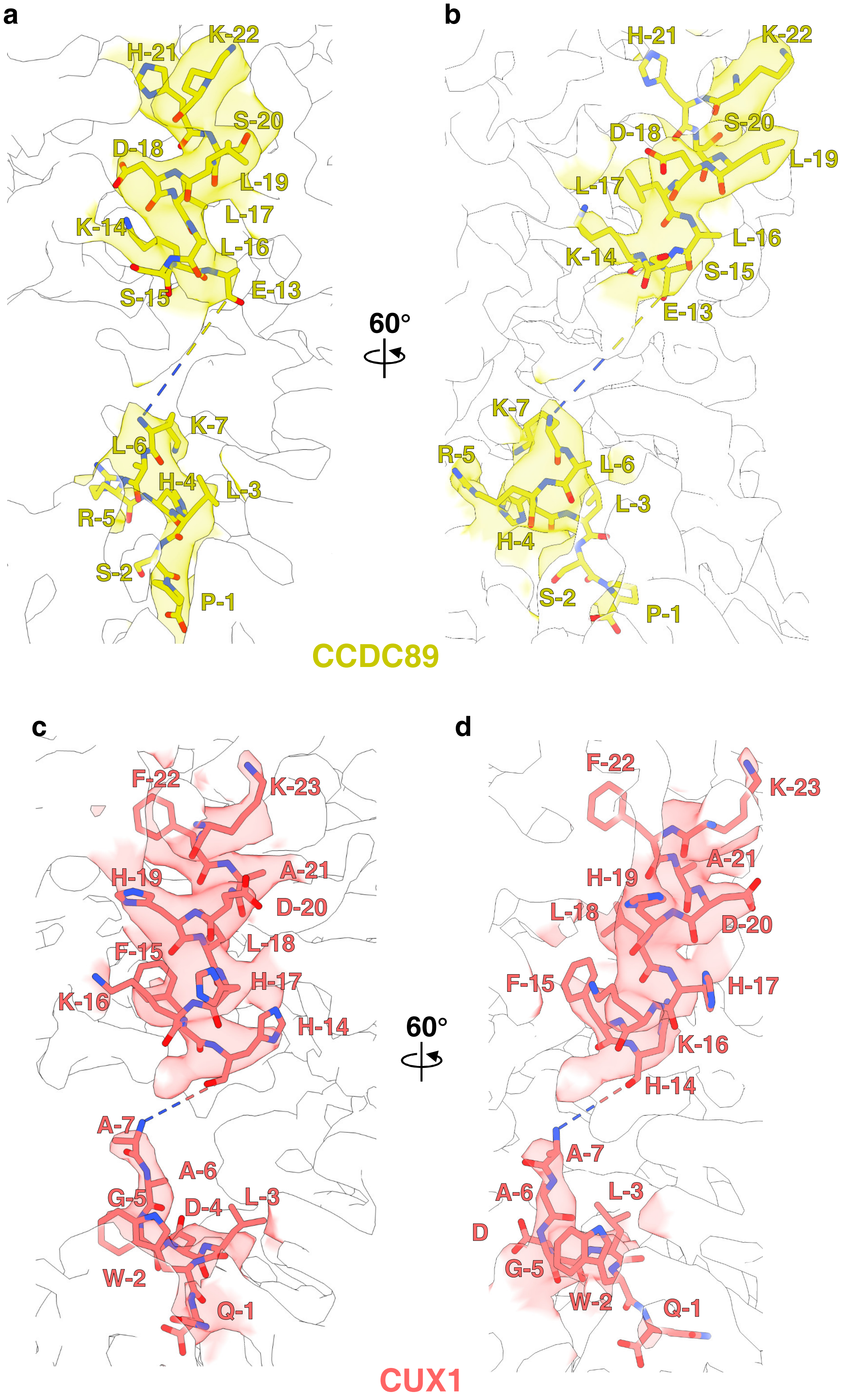

Supplementary Figure 11. The cryo-EM maps of the C-degron peptides in the CRL2^{FEM1B} structures. a-b The cryo-EM map of CCDC89 peptide ($^{-22}$KHSLDLLSKERELNGKLRHLSP^{-1}) shown in yellow; **c-d** The cryo-EM map of CUX1 peptide ($^{-23}$KFADHLHKFHENDNGAAAGDLWQ^{-1}) shown in salmon. The invisible fragments are indicated by dashes.

Supplementary Figure 11

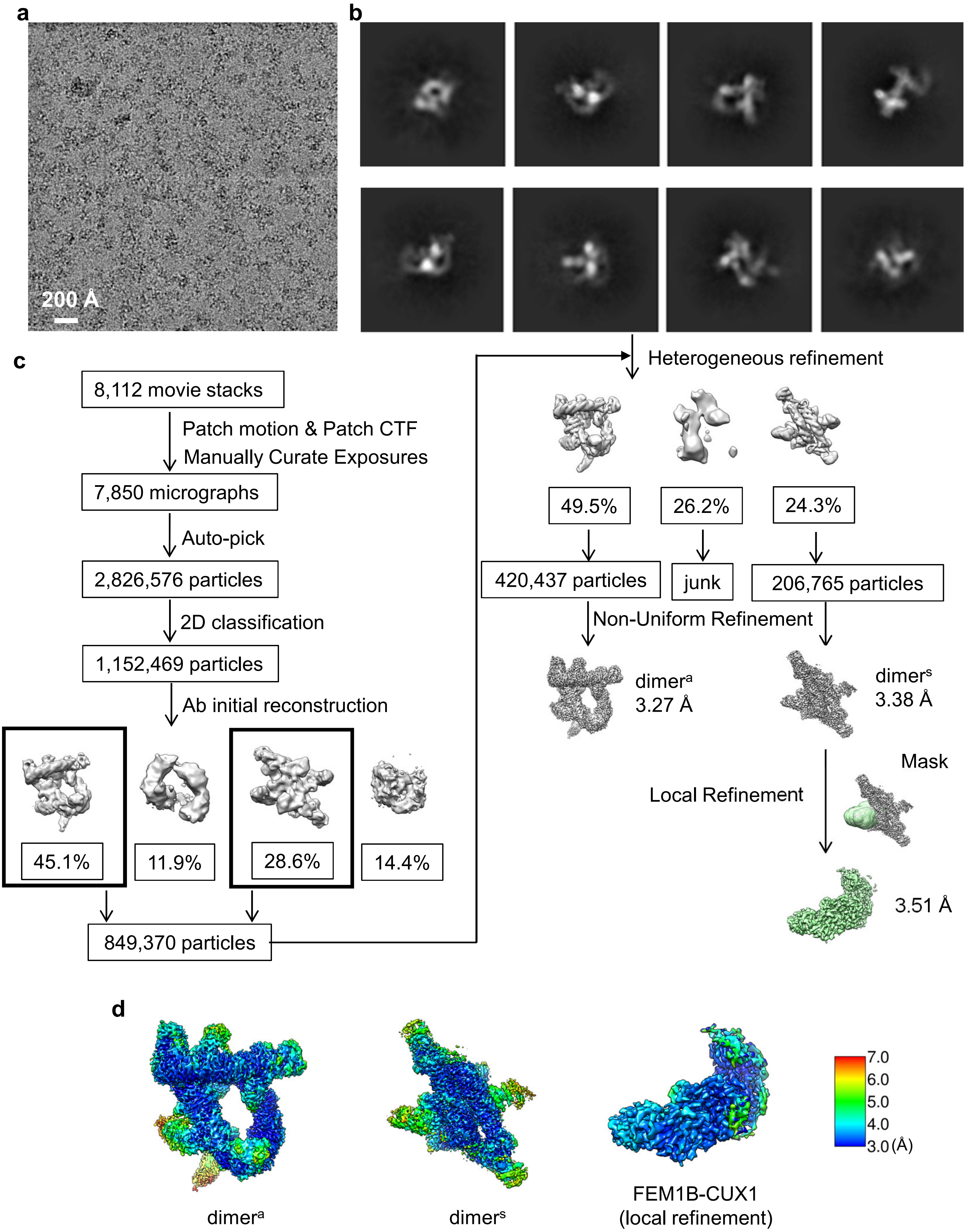

Supplementary Figure 12

e

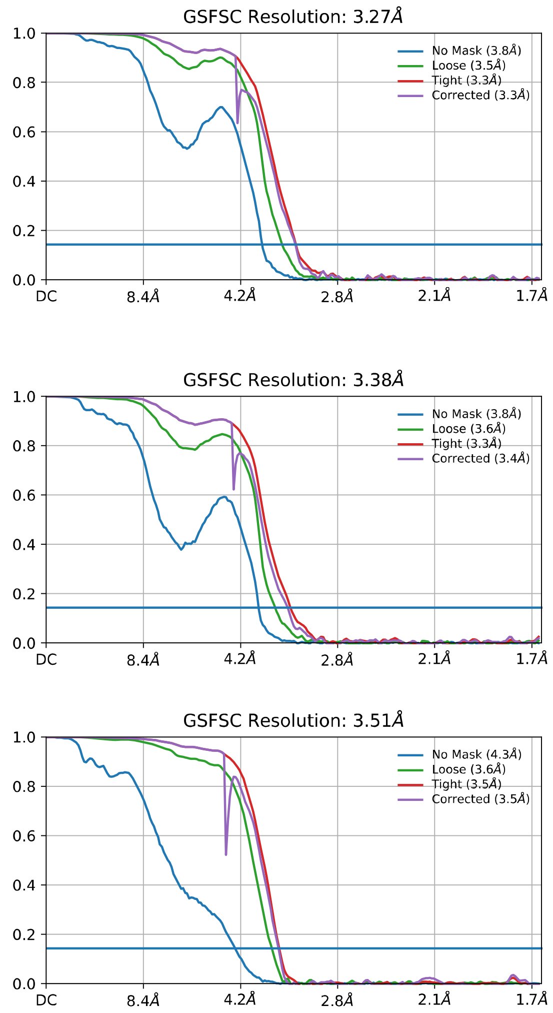

f

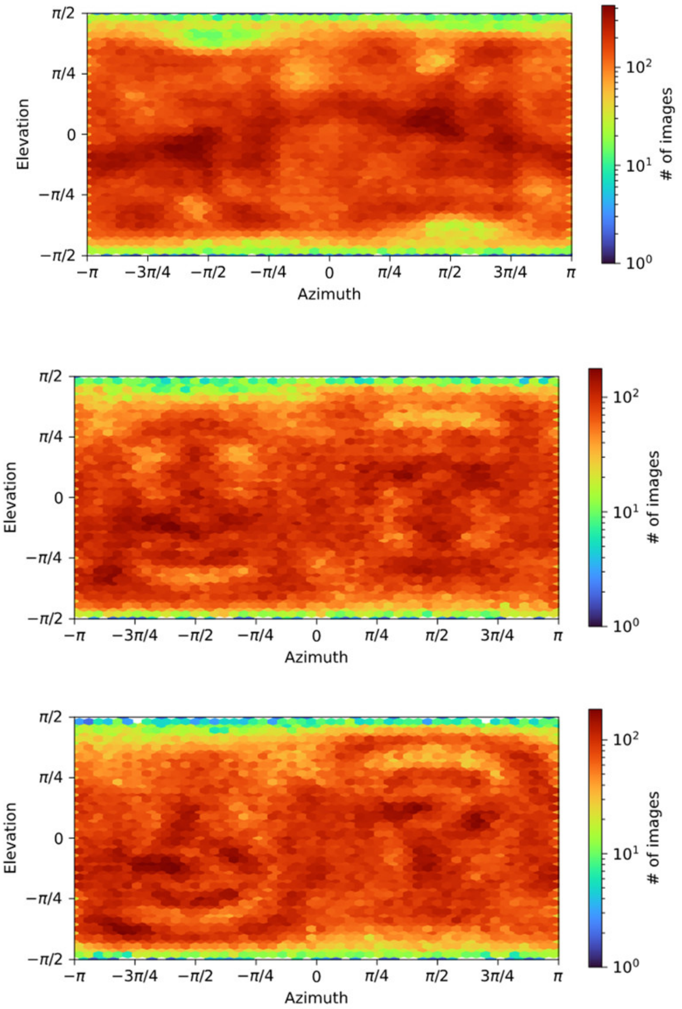

Supplementary Figure 12. Single-particle cryo-EM analysis of the neddylated CRL2^{FEM1B} E3 ligase complex with the CUX1 C-degron. **a** Representative motion-corrected cryo-EM micrograph. **b** Reference-free 2D class averages. **c** Workflow of the data processing. **d** Resolution maps for the final 3D reconstructions of dimer^{a} (left), dimer^{s} (middle), and the FEM1B-CUX1 complex (right). **e** Gold standard FSC plots for the 3D reconstructions of dimer^{a} (upper), dimer^{s} (middle), and the FEM1B-CUX1 complex (bottom), calculated in cryoSPARC. **f** Euler angle distribution of the particle images.

Supplementary Figure 12

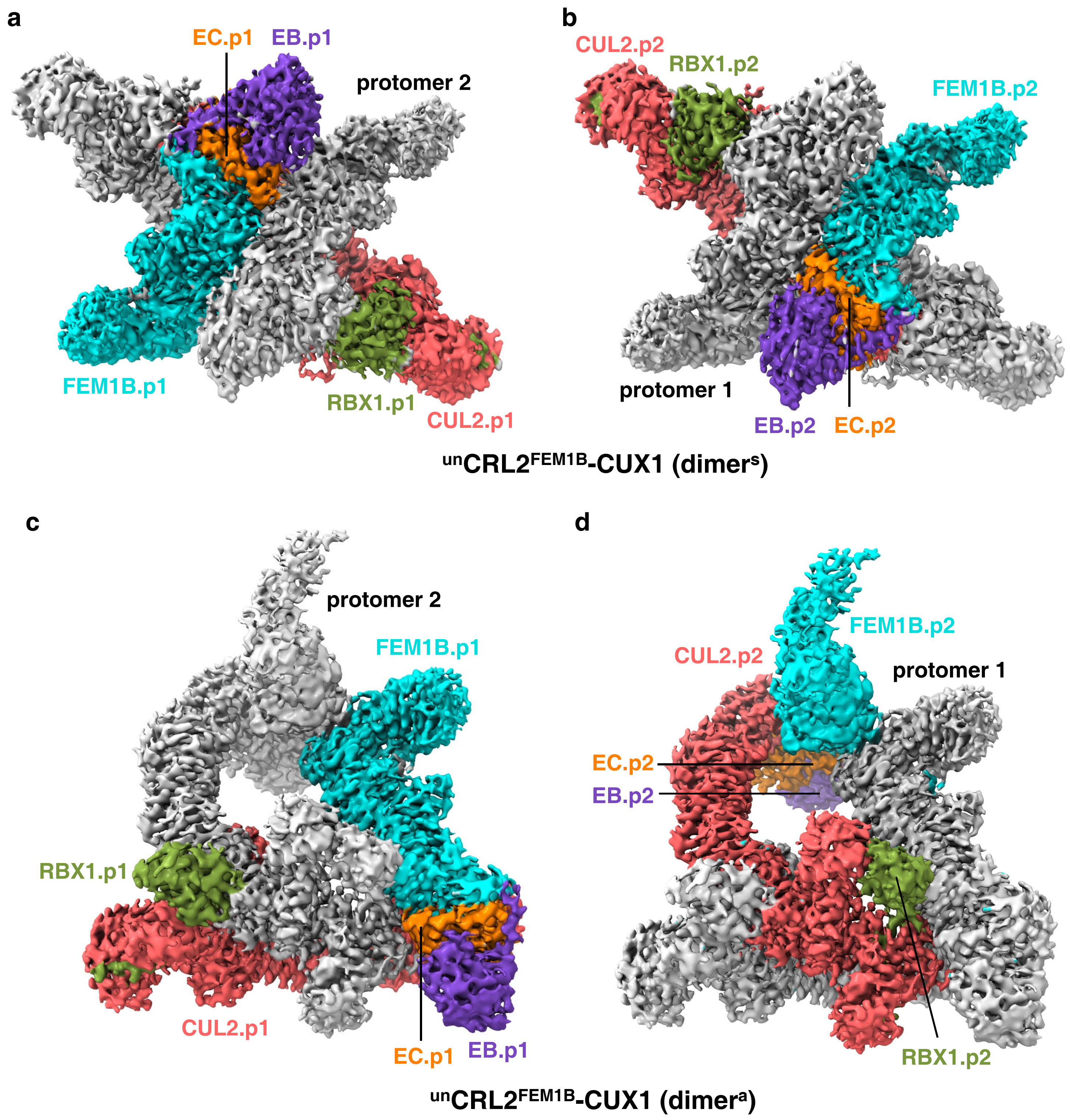

Supplementary Figure 13. The maps of the two dimer states of ^{un}CRL2^{FEM1B} bound with the CUX1 C-degron. **a-b** Maps of the ^{un}CRL2^{FEM1B} dimer^{s} bound with the CUX1 C-degron, with protomer 1 colored by subunits and protomer 2 colored in grey (a), or protomer 2 colored by subunits and protomer 1 colored in grey (b). **c-d** Maps of the ^{un}CRL2^{FEM1B} dimer^{a} bound to the CUX1 C-degron, with protomer 1 colored by subunits and protomer 2 in grey (c), or protomer 2 colored by subunits and protomer 1 in grey (d).

Supplementary Figure 13

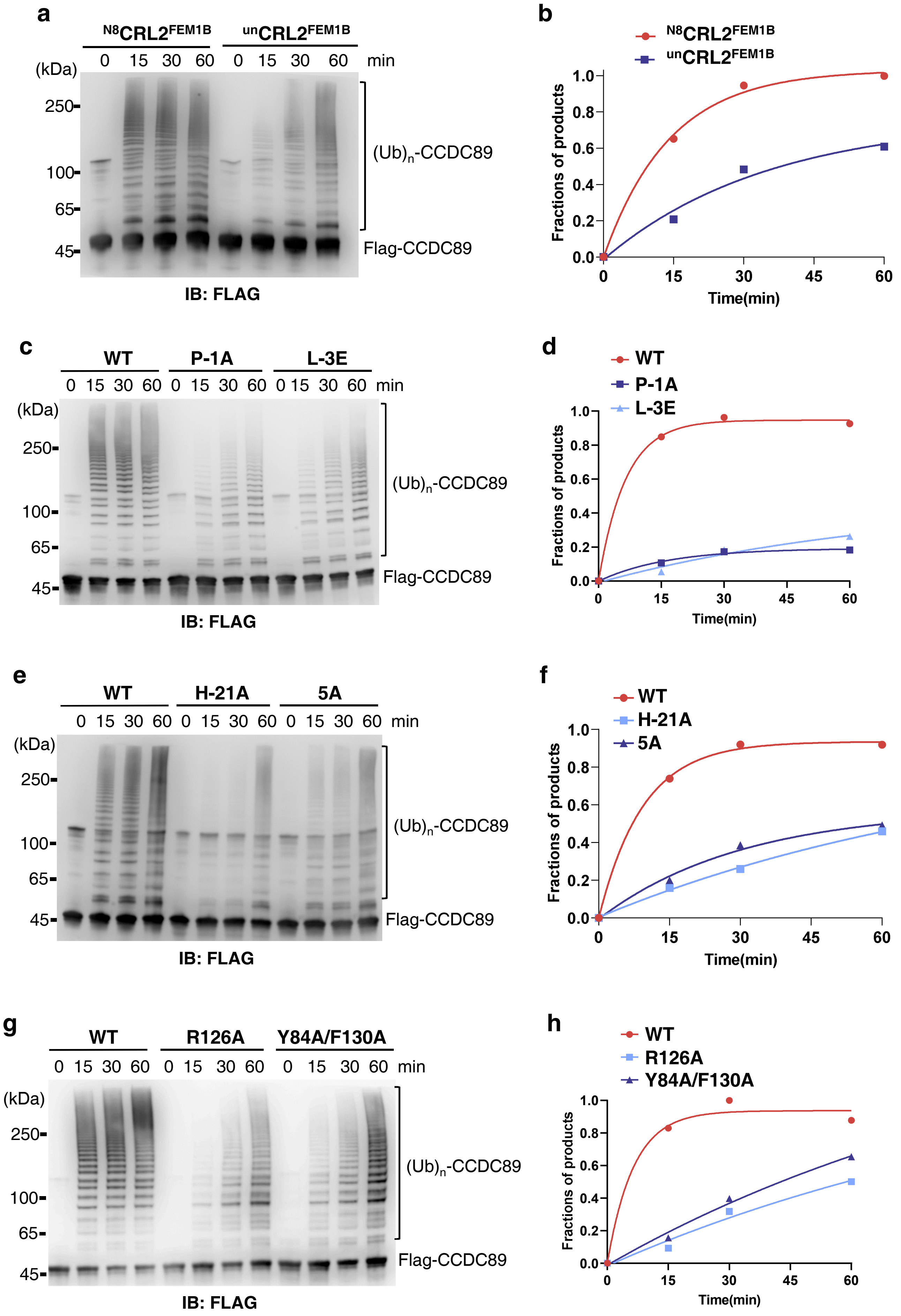
a
N8CRL2FEM1B
unCRL2FEM1B
0 15 30 60 0 15 30 60 min
(kDa)
250
100
65
45
(Ub)n-CCDC89
Flag-CCDC89
IB: FLAG
b
N8CRL2FEM1B
unCRL2FEM1B
Fractions of products
1.0
0.8
0.6
0.4
0.2
0.0
0 15 30 45 60
Time(min)
c
WT
P-1A
L-3E
0 15 30 60 0 15 30 60 0 15 30 60 min
(kDa)
250
100
65
45
(Ub)n-CCDC89
Flag-CCDC89
IB: FLAG
d
WT
P-1A
L-3E
Fractions of products
Time(min)
e
WT
H-21A
5A
0 15 30 60 0 15 30 60 0 15 30 60 min
(kDa)
250
100
65
45
(Ub)n-CCDC89
Flag-CCDC89
IB: FLAG
f
WT
H-21A
5A
Fractions of products
Time(min)
g
WT
R126A
Y84A/F130A
0 15 30 60 0 15 30 60 0 15 30 60 min
(kDa)
250
100
65
45
(Ub)n-CCDC89
Flag-CCDC89
IB: FLAG
h
WT
R126A
Y84A/F130A
Fractions of products
Time(min)

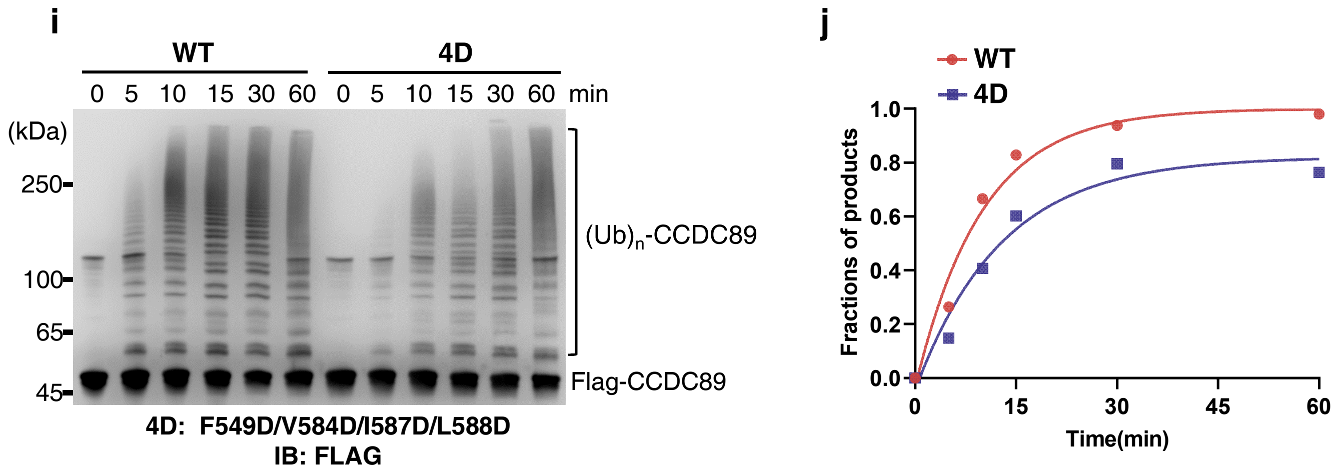

Supplementary Figure 14. *In vitro* ubiquitination assay to dissect the FEM1B function toward the CCDC89 C-degron. In combination with E1 and E2, CRL2^{FEM1B} and its variants catalyzes the polyubiquitination of His-FLAG-GFP-fusion of CCDC89 and its mutants. *In vitro* assays of: **a-b** ^{N8}CRL2^{FEM1B} and ^{un}CRL2^{FEM1B} with WT CCDC89. **c-d** ^{N8}CRL2^{FEM1B} with CCDC89 WT and two single mutants, P-1A and L-3E. **e-f** ^{N8}CRL2^{FEM1B} with CCDC89 WT and the H-21A mutant, and the ^{N8}CRL2^{FEM1B} 5A mutant with CCDC89. **g-h** ^{N8}CRL2^{FEM1B} WT, R126A and Y84A/F130A mutants with CCDC89. **i-j** N8-CRL2^{FEM1B} and 4D mutant with CCDC89. All experiments were repeated three times with similar results. Source data are provided as a Source Data file.

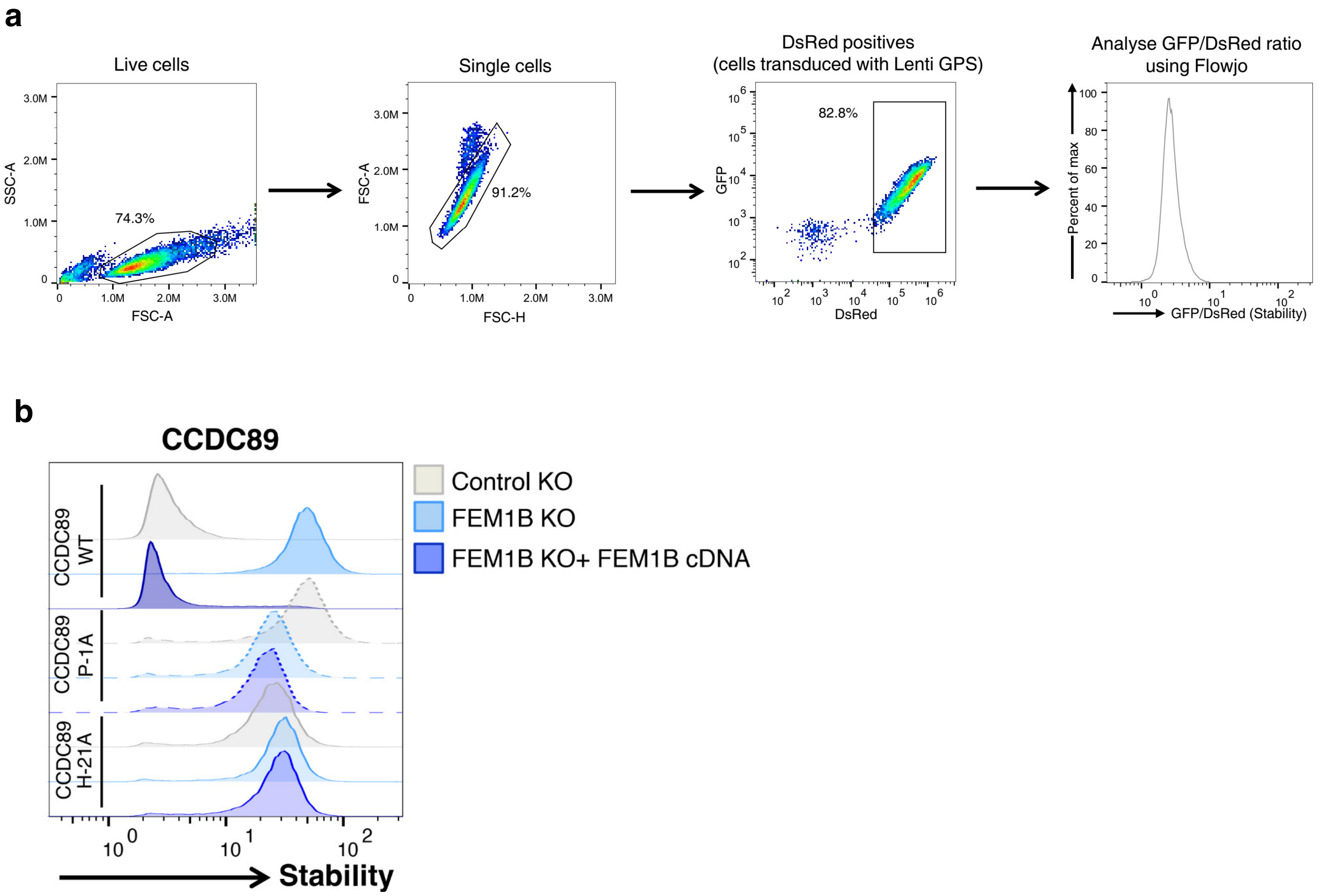

Supplementary Figure 15. *In vivo* GPS assay to dissect FEM1B function toward C-degrons. a Gating strategy for flow cytometry. Live cells were gated based on FSC-A vs. SSC-A; single cells were gated using FSC-H vs. FSC-W; DsRed positive cells (containing the lentiviral integrated GPS reporters) were used to analyse the GFP/DsRed ration that reflect the stability of the GFP-fused substrate. **b** Stability analysis as analyzed by flow cytometry of the 23-aa CCDC89 C-degron WT and mutants in FEM1B KO cells rescued with FEM1B cDNA. Cells transfected with sgRNA targeting AAVSI (Control KO) serve as the reference of substrate instability.

Supplementary Figure 15

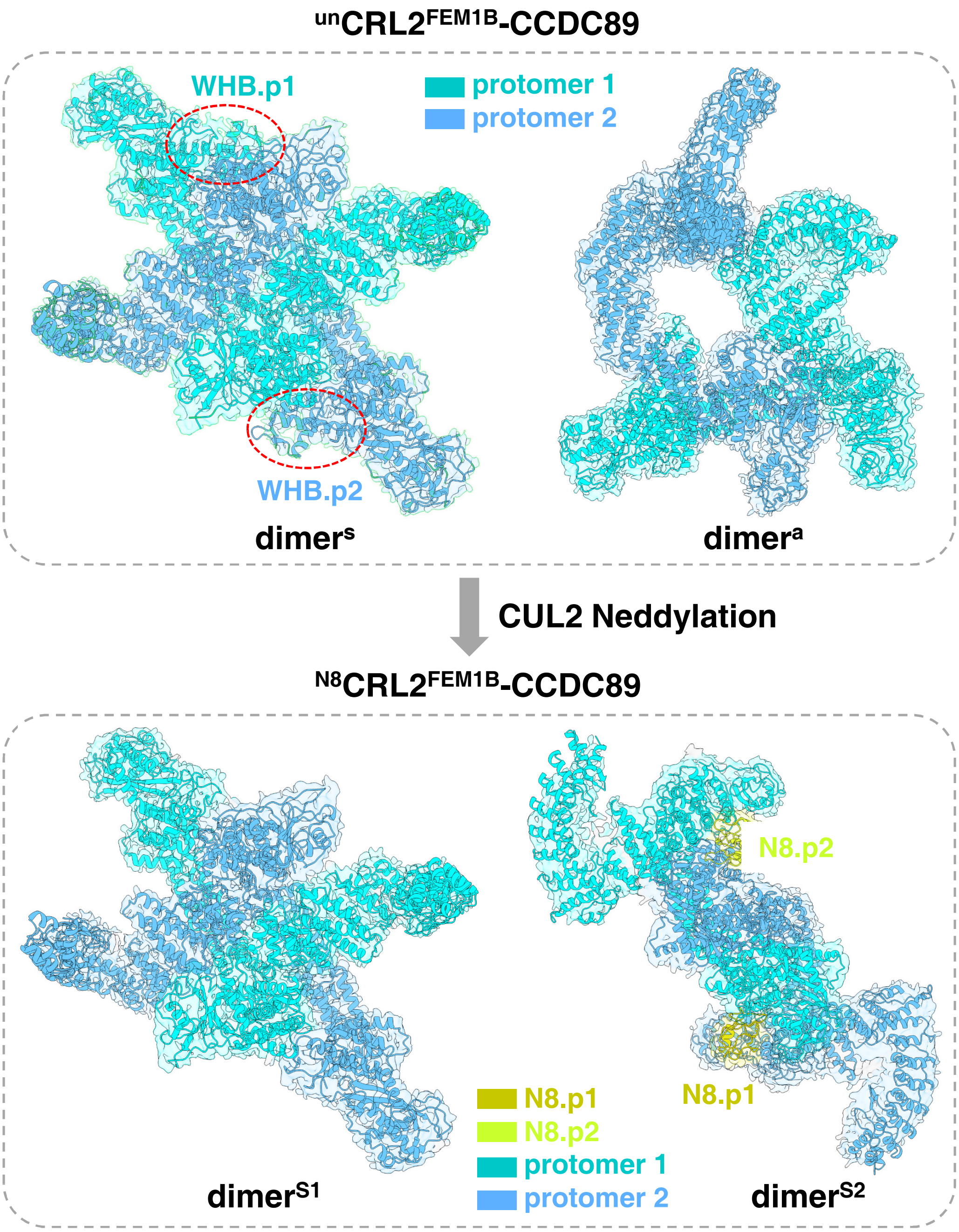

Supplementary Figure 16. The dimerization assembly of ^{un}CRL2^{FEM1B}-CCDC89 and ^{N8}CRL2^{FEM1B}-CCDC89. In all dimer structures, the two protomers were colored by cyan and blue with maps. Upon CUL2 neddylation, the two NEDD8 molecules, N8.p1 and N8.p2, were colored with maps. The WHB.p1 and WHB.p2 are highlighted in the structure of unCRL2^{FEM1B}-CCDC89 dimer^{s}

Supplementary Figure 16

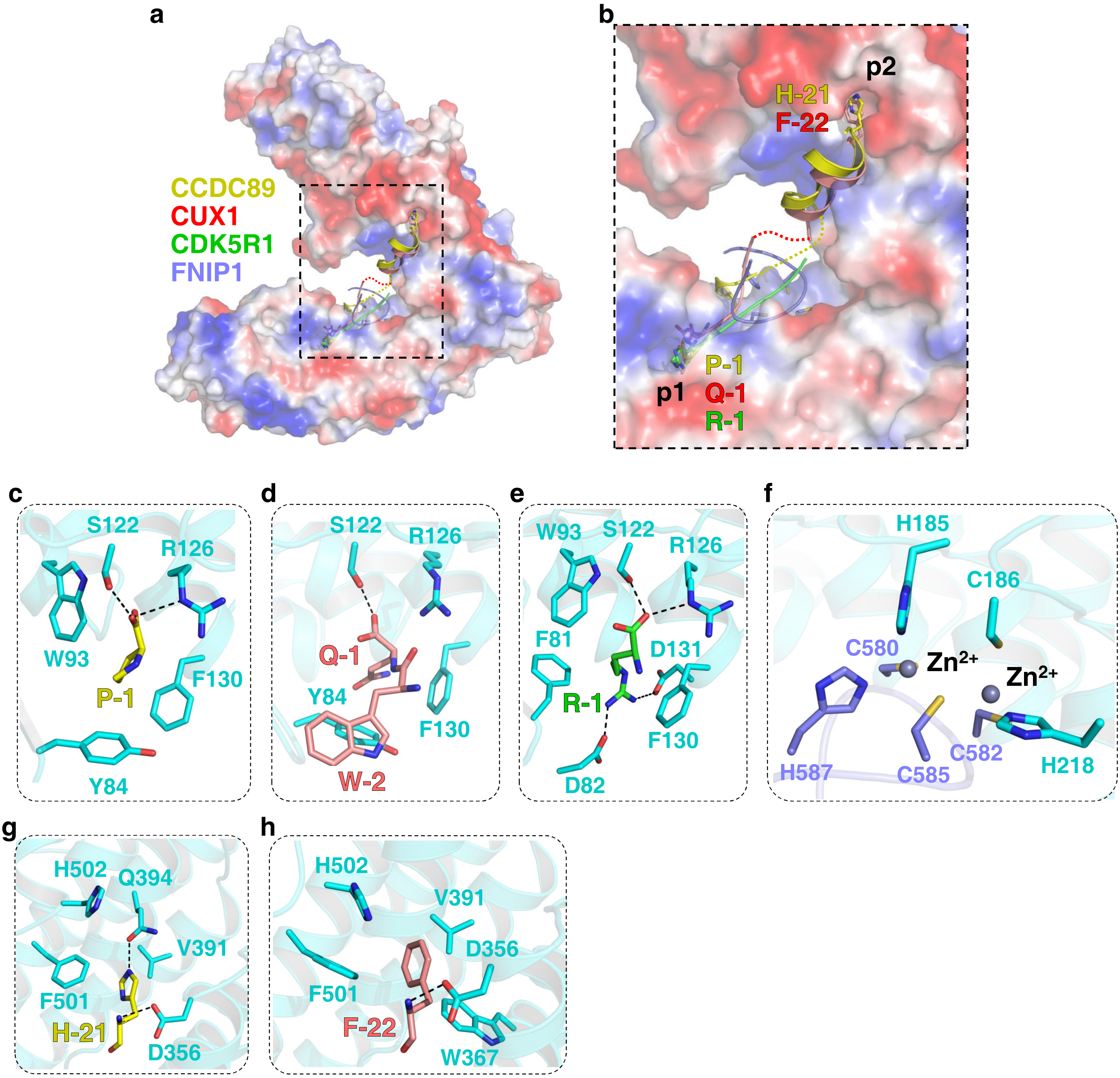

Supplementary Figure 17. Comparing the FEM1B-degron interaction modes. **a** Superimposed structures of FEM1B-CCDC89, FEM1B-CUX1, FEM1B-CDK5R1 (PDB: 7CNG), and FEM1B-FNIP1 (PDB: 7ROY). The electrostatic surface of FEM1B is shown bound with the degron peptides, which are shown in cartoon. **b** close-up view of the degrons bound on the FEM1B surface. **c** Interactions between FEM1B (cyan) and CCDC89 Pro-1 (yellow). **d** Interactions between FEM1B (cyan) and Gln-1 and Trp-2 of CUX1 (salmon). **e** Interactions between FEM1B (cyan) and CDK5R1 Arg-1 (green). **f** Zn^{2+}-mediated interactions between FEM1B (cyan) and FNIP1 (purple). **g** Interactions between FEM1B (cyan) and CCDC89 His-21 (yellow). **h** Interactions between FEM1B (cyan) and CUX1 Phe-22 (salmon).

Supplementary Figure 17

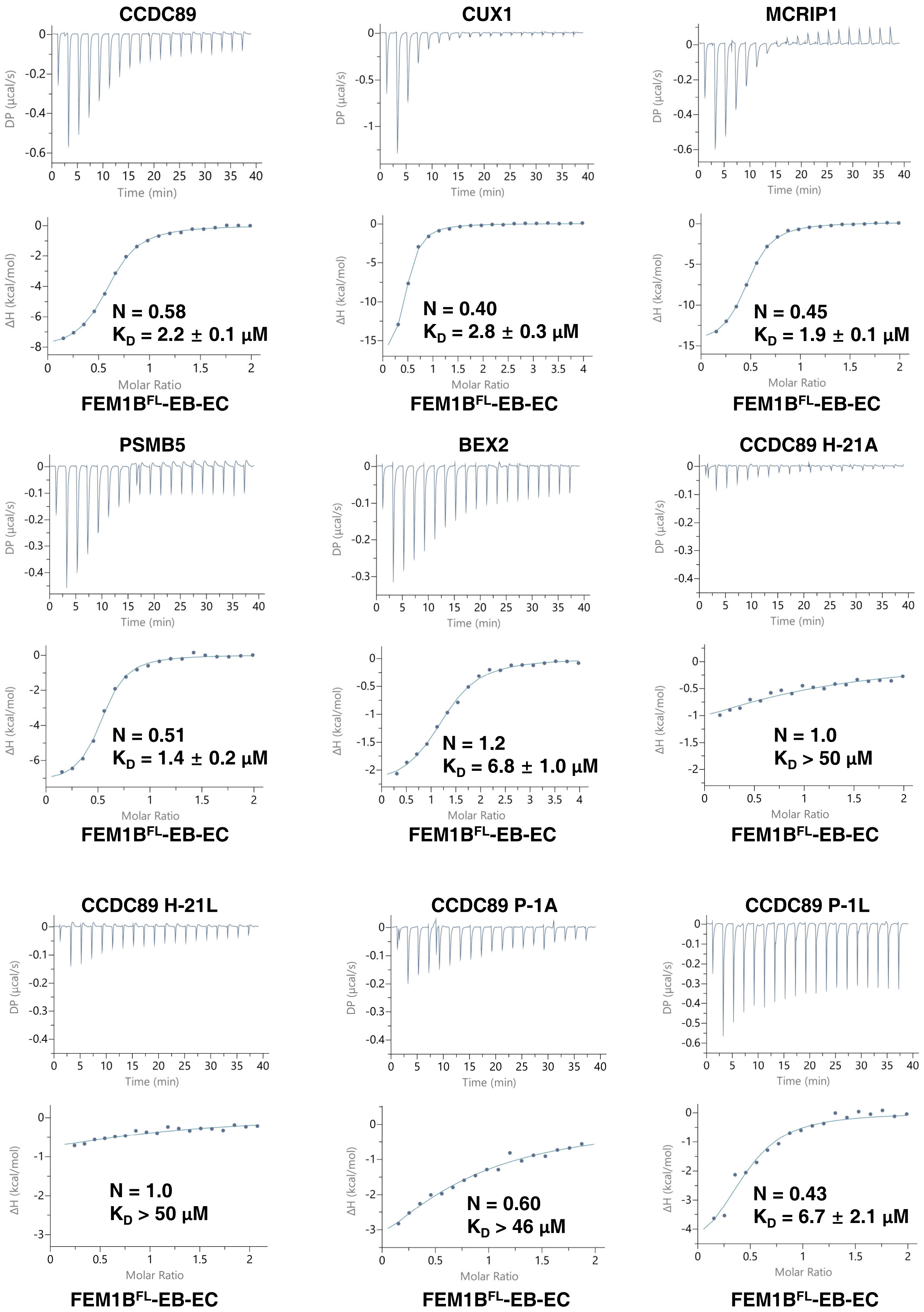

Supplementary Figure 18

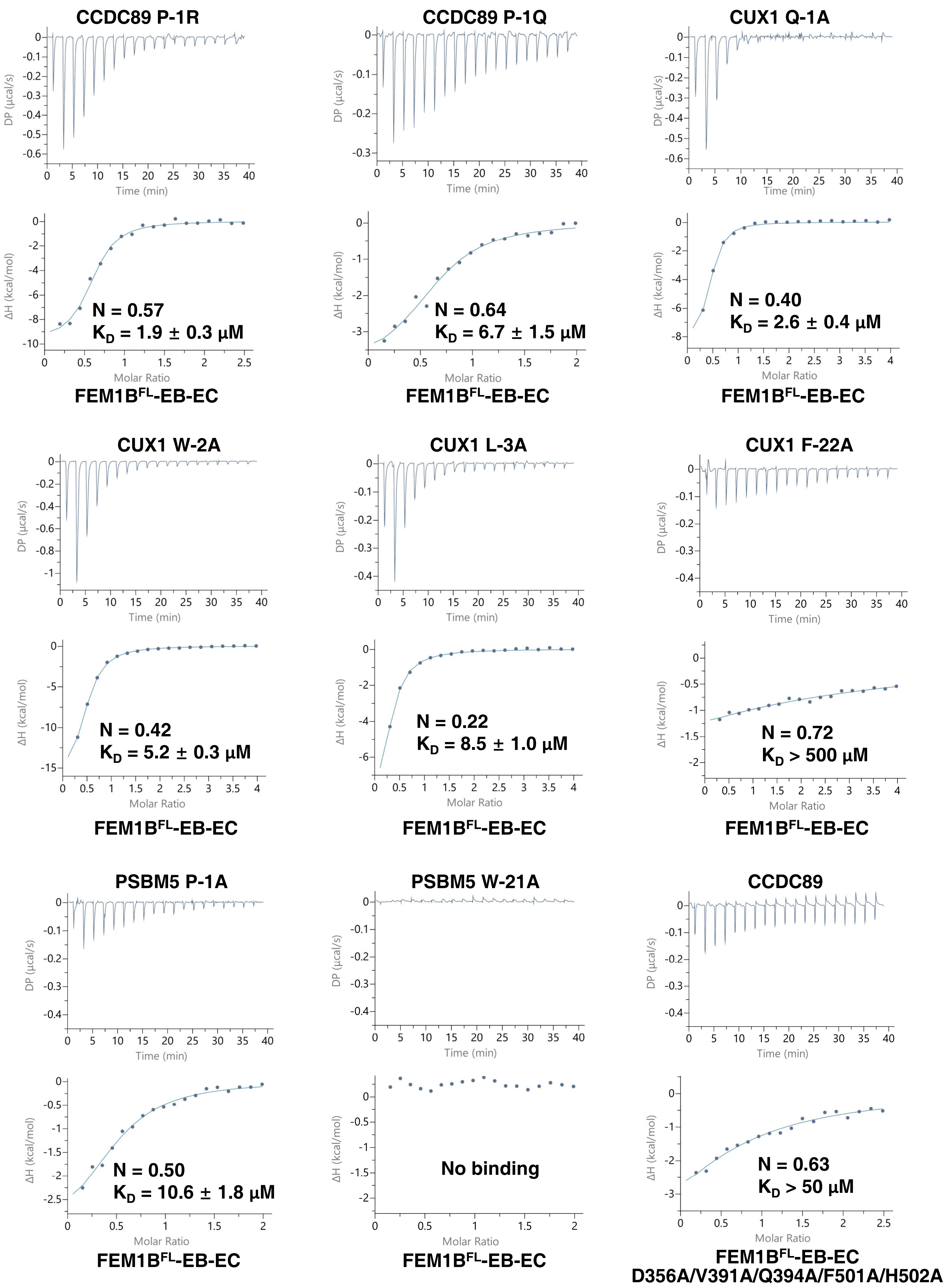

Supplementary Figure 18

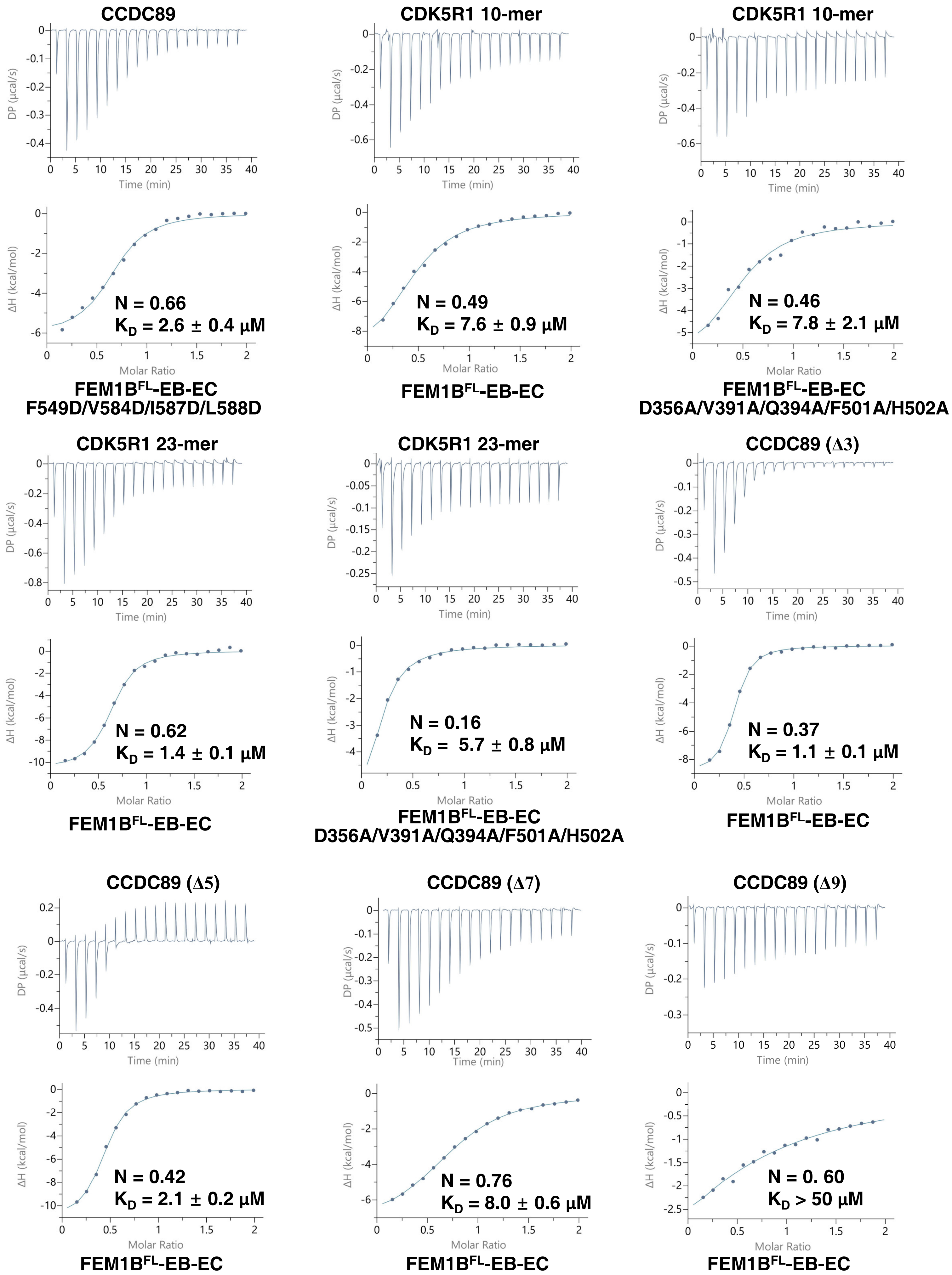

Supplementary Figure 18

Supplementary Figure 18. ITC curves for the FEM1B^{FL}-EB-EC variants binding to different C-degrons.

Supplementary Figure 18

Supplementary Table 1. Cryo-EM data collection, refinement and validation statistics

	^{un}CRL2^{FEM1B}-CCDC89 symmetric dimer	^{un}CRL2^{FEM1B}-CCDC89 asymmetric dimer	^{N8}CRL2^{FEM1B}-CCDC89 dimer^{S1}	^{N8}CRL2^{FEM1B}-CCDC89 dimer^{S2}	^{N8}CRL2^{FEM1B}-CDK5R1 dimer^{s2}
**Data collection and processing**					
Microscope	Titan Krios	Titan Krios	Titan Krios	Titan Krios	Titan Krios
Magnification	105,000×	105,000×	105,000×	105,000×	105,000×
Voltage (kV)	300	300	300	300	300
Exposure rate (e^-/ Å^2)	57.6	57.6	57.6	57.6	57.6
Defocus range (μm)	-1.5 − -2.9	-1.5 − -2.9	-1.5 − -2.9	-1.5 − -2.9	-1.5 − -2.9
Pixel size (Å)	0.82	0.82	0.82	0.82	0.82
Symmetry imposed	C1	C1	C2	C1	C1
Initial particle images (no.)	1,577,333	1,577,333	999,541	999,541	1,180,118
Final particle images (no.)	470,742	236,621	84,450	102,432	436,966
Map resolution range (Å)	3.0-7.0	3.0-7.0	3.0-7.0	3.0-7.0	3.0-7.0
Model resolution (Å)	3.39	3.37	3.44	4.09	3.54
FSC threshold	0.143	0.143	0.143	0.143	0.143
**Model composition**					
Nonhydrogen atoms	26476	26255	24811	26840	26848
Protein residues	3301	3265	3091	3395	3397
**Validation**					
Bonds (RMSD)					
Lengths (Å)	0.003	0.003	0.003	0.003	0.003
Angles (°)	0.64	0.64	0.54	0.60	0.58
MolProbity score	1.83	1.80	1.81	1.99	1.89
Clashscore	9.42	7.33	8.42	11.01	9.61
Poor rotamers (%)	0	0	0	0	0
Ramachandran plot					
Favored (%)	95.2	94.0	94.9	93.4	94.4
Allowed (%)	4.8	6.0	5.1	6.6	5.6
Disallowed (%)	0	0	0	0	0

Supplementary Table 1. Cryo-EM data collection, refinement and validation statistics

	FEM1B^{FL}-CCDC89 local refinement	^{un}CRL2^{FEM1B}-CUX1 symmetric dimer	^{un}CRL2^{FEM1B}-CUX1 asymmetric dimer	FEM1B^{FL}-CUX1 local refinement
**Data collection and processing**				
Microscope	Titan Krios	Titan Krios	Titan Krios	Titan Krios
Magnification	105,000×	105,000×	105,000×	105,000×
Voltage (kV)	300	300	300	300
Exposure rate (e^{-}/ Å^{2})	57.6	57.6	57.6	57.6
Defocus range (μm)	-1.5 − -2.9	-1.5 − -2.9	-1.5 − -2.9	-1.5 − -2.9
Pixel size (Å)	0.82	0.82	0.82	0.82
Symmetry imposed	C1	C1	C1	C1
Initial particles (no.)	999,541			
Final particles (no.)	84,450			
Map resolution range (Å)	3.0-7.0	3.0-7.0	3.0-7.0	3.0-7.0
Map resolution (Å)	3.55	3.38	3.27	3.51
FSC threshold	0.143	0.143	0.143	0.143
**Model composition**				
Nonhydrogen atoms	5060	26311	23404	5074
Protein residues	644	3276	2901	644
**Validation**				
Bonds (RMSD)				
Lengths (Å)	0.003	0.003	0.004	0.003
Angles (°)	0.58	0.56	0.60	0.62
MolProbity score	1.87	1.81	1.86	2.05
Clashscore	8.11	8.45	8.00	9.29
Poor rotamers (%)	1.09	0	0	0
Ramachandran plot				
Favored (%)	94.0	94.9	93.6	89.7
Allowed (%)	6.0	5.1	6.4	10.3
Disallowed (%)	0	0	0	0

Supplementary Table 2. The sequences of SUMO-fusion peptides used for ITC binding experiments

**Peptide names**	**Sequences of SUMO fusion peptide**
$CCDC89_{352-374}$ WT	SUMO-$(GGGS)_2$-KKHSLDLLSKERELNGKLRHLSP
$CCDC89_{352-374}$ (P-1A)	SUMO-$(GGGS)_2$-KKHSLDLLSKERELNGKLRHLSA
$CCDC89_{352-374}$ (P-1R)	SUMO-$(GGGS)_2$-KKHSLDLLSKERELNGKLRHLSR
$CCDC89_{352-374}$ (P-1L)	SUMO-$(GGGS)_2$-KKHSLDLLSKERELNGKLRHLSL
$CCDC89_{352-374}$ (P-1Q)	SUMO-$(GGGS)_2$-KKHSLDLLSKERELNGKLRHLSQ
$CCDC89_{352-374}$ (H-21A)	SUMO-$(GGGS)_2$-KKASLDLLSKERELNGKLRHLSP
$CCDC89_{352-374}$ (H-21L)	SUMO-$(GGGS)_2$-KKLSLDLLSKERELNGKLRHLSP
$CCDC89_{352-374}$ Δ3	SUMO-$(GGGS)_2$-KKHSLDLLSKEREL~~NGK~~LRHLSP
$CCDC89_{352-374}$ Δ5	SUMO-$(GGGS)_2$-KKHSLDLLSKER~~ELNGK~~LRHLSP
$CCDC89_{352-374}$ Δ7	SUMO-$(GGGS)_2$-KKHSLDLLSK~~ERELNGK~~LRHLSP
$CCDC89_{352-374}$ Δ9	SUMO-$(GGGS)_2$-KKHSLDLL~~SKERELNGK~~LRHLSP
$CUX1_{656-678}$ WT	SUMO-$(GGGS)_2$-KFADHLHKFHENDNGAAAGDLWQ
$CUX1_{656-678}$ (Q-1A)	SUMO-$(GGGS)_2$-KFADHLHKFHENDNGAAAGDLWA
$CUX1_{656-678}$ (W-2A)	SUMO-$(GGGS)_2$-KFADHLHKFHENDNGAAAGDLAQ
$CUX1_{656-678}$ (L-3A)	SUMO-$(GGGS)_2$-KFADHLHKFHENDNGAAAGDAWQ
$CUX1_{656-678}$ (F-22A)	SUMO-$(GGGS)_2$-KAADHLHKFHENDNGAAAGDLWQ
$PSMB5_{241-263}$ WT	SUMO-$(GGGS)_2$-DGWIRVSSDNVADLHEKYSGSTP
$PSMB5_{241-263}$ (P-1A)	SUMO-$(GGGS)_2$-DGWIRVSSDNVADLHEKYSGSTA
$PSMB5_{241-263}$ (W-21A)	SUMO-$(GGGS)_2$-DGAIRVSSDNVADLHEKYSGSTP
MCRIP1	SUMO-$(GGGS)_2$-GWTIDMRRNEPERTRSLSAPGPL
$BEX2_{116-128}$ WT	SUMO-$(GGGS)_2$-HSLRAVSTDPPHHDHHDEFCLMP
$CDK5R1_{298-307}$ WT	SUMO-$(GGGS)_2$-KKRLLLGLDR
$CDK5R1_{285-307}$ WT	SUMO-$(GGGS)_2$-VFSDLKNESGQEDKKRLLLGLDR

Supplementary Table 3. Primers used for expression of the APPBP2 mutants in mammalian cell.

Primer names	Sequences of primers
Y84A_F	ggcaccatcaatgacagccccgtcgaagcggacg
Y84A_R	cgtccgcttcgacggggctgtcattgatggtgcc
R126A_F	CTAATTCAACCCCCCTGgcgGCAGCATGCTTTGATG
R126A_R	CATCAAAGCATGCTGCcgcCAGGGGGGTTGAATTAG
F130A_F	CTGCGGGCAGCATGCgctGATGGCAGACTGGA
F130A_R	TCCAGTCTGCCATCagcGCATGCTGCCCGCAG
F549D_F	gatggagtgcaaggtcaaatcatcactgatgggcctgttg
F549D_R	caacaggcccatcagtgatgatttgaccttgcactccatc
V584D/I587D/L588D_F	ggcacttgagactcatcttcatttgagttttaagatcatcttcagaatccccagttgtacttttgtc tagcggagtcttatt
V584D/I587D/L588D_R	aataagactccgctagacaaaagtacaactggggattctgaagatgatcttaaaactcaaat gaagatgagtctcaagtgcc
D356A_F	ctgctcaaattccatattagccgcataaacagctcctct
D356A_R	agaggagctgtttatgcggctaatatggaatttgagcag
V391A/Q394A_F	caaggatcttcttcgatttgctcaagctttctcagcaatgatacatttgaatgaaactgtga
V391A/Q394A_R	tcacagtttcattcaaatgtatcattgctgagaaagcttgagcaaatcgaagaagatccttg
F501A/H502A_F	ctgtcaattccaatactccagttgatgatgccgccaccaatgacgtctgc
F501A/H502A_R	gcagacgtcattggtggcggcatcatcaactggagtattggaattgacag
